# London Sewage

**Published:** 1900-03-17

**Authors:** 


### London Sewage.

The discourse given last Friday by Dr. Frank Clowes at the Royal Institution on "Bacteria and Sewage" put into form the most recent results arrived at in regard to this interesting and important subject. For the past two years Dr. Clowes and Dr. Houston have been carrying out experiments on behalf of the London County Council, and seeing that ever since the year 1893 a large area has been in constant use for experimental purposes at the Barking sewer outfall, it would appear that any conclusions now arrived at have reached a stage beyond theory, and may be taken as representing much practical experience. As is well known, there are many methods by which most of the difficulties connected with irrigation by crude sewage may be obviated, and by which the amount of land required for the production of a clean effluent can be very materially diminished. Many towns, however, and London is one, are so situated that they find it extremely difficult, if not impossible, to utilise their sewage by any form of irrigation. The height of their ambition is to render the effluent sufficiently innocuous to allow of its being poured out into the natural water courses, and in regard to the possibility of attaining this end by a comparatively simple process, and at a comparatively small expense, the results arrived at by Dr. Clowes are of much importance.

The method which has been adopted for some years back at the Barking outfall has been, after the raw sewage has been roughly screened and subjected to a rapid process of sedimentation, in order to remove sand, woody fibre, &c., to run it into a bed of coke an acre in extent and from three to four feet thick, allow it to stand for three hours, and then withdraw the liquid, which after this simple treatment is found to be entirely free from offensive odour. The purification thus effected in the coke-beds depends on the action of bacteria by which the coke surfaces quickly become coated, the alternate supply of oxygen and sewage leading to the more or less complete oxidisation of the decomposable material contained in the latter. Dr. Clowes says that on an average 51·3 per cent. of the dissolved matter of the original sewage is thus removed, by which we may suppose that he means so far oxidised as to form a stable combination, and that by repeating the process a still larger proportion can be got rid of. More recently, however, he has been experimenting with a bed of coke thirteen feet thick, which has proved even more efficient, the fluid flowing from it being quite inoffensive, and remaining so even after it had been kept for a month. This effluent, he said, was well aërated, and remained so, having enough dissolved oxygen to support the life of fish, which, indeed, had been kept in it for a month without detriment to their health. Now it must be admitted that to pour away such an effluent, containing as it must do a large amount of assimilable and most useful nutrient salts, is a wasteful proceeding. Far better where there is land make use of it for irrigating purposes. The investigations recently made by Dr. Houston, moreover, go to show that both in crude sewage, in the slime deposited in the coke-beds and also in the effluent, a considerable number of microbes exist which are distinctly fatal to small animals, and are probably closely allied to ordinary pathogenic micro-organisms, so that we must all agree with the position taken up by the advisers of the Local Government Board, that where an effluent has to enter a river which may have to supply drinking-water to places lower down the stream no process of purification should be deemed acceptable which does not include land irrigation. But the matter is somewhat different in regard to London, whose sewage discharges into a tidal river. Here it is a mere matter of cleanliness and economy, and Dr. Clowes maintained that the introduction of such an effluent as he described into the lower reaches of the Thames would be unobjectionable, since the water there could not in any case be used as for drinking purposes. Such an effluent, he said, would certainly cause no deposit on the river bed, and would even tend to clarify the muddy river water; it would cause no smell, and it would be suitable for the support of healthy fish life. We think he is right under the limitation of the case. Certainly the present system, although almost surprisingly efficient, is far from ideal. The storage of the sewage, its chemical treatment by lime and by iron sulphate, its sedimentation in vast underground chambers, the pumping of the sludge so precipitated into great steamships, its transport to the Nore, and then the casting of it out as food for fishes, is from end to end an unsatisfactory and makeshift proceeding, and it is to be hoped that before long we may see something better in operation.

